# The difference distance between the apical foramen and the anatomical apex in primary teeth—An in vitro study

**DOI:** 10.1002/cre2.784

**Published:** 2023-09-13

**Authors:** Ansam Shafik Alafandy, Ramah Eimad Makieh

**Affiliations:** ^1^ Pedodontics Department Arab International University Daraa Syrian Arab Republic; ^2^ Pediatric Dentistry Department Damascus University Damascus Syrian Arab Republic

**Keywords:** anatomical apex, apical foramen, difference distance and primary teeth

## Abstract

**Introduction:**

The apex area in the primary teeth changes continuously due to the physiologic resorption, therefore; the apical foramen (AF) may not correspond to the anatomic apex (AA), which gives a big challenge to achieve successful endodontic treatment. The aim of this research was to study the difference distance (DD) between the position of the AA and AF, besides the difference acceptance (DA) in primary teeth, and the effect of the following variables: root canal curvature, resorption degree, and canal size on DD and DA separately.

**Methods:**

In this research, 180 root canals from 60 primary teeth were studied. Two lengths of each canal were measured by a K‐file from a certain point in the crown; the first length was until the AA and the second was until the AF. Then DD was obtained by calculating the difference between those two lengths. Statistical analysis tests were done. A *p* value of <.05 was considered significant at a 95% confidence level.

**Results:**

The percentage of canals with 0 mm DD was 34.4%, while it was 1.1% with DD of 6 mm. The percentages of acceptable ( ≤ 2 mm) and unacceptable ( > 2 mm) difference were 84.4% and 15.6%, respectively. There was a significant difference in the DD value between the three groups of curvature degree and the three groups of canal size. There was a significant difference between the DA in the three groups of canal size.

**Conclusion:**

DD has a wide variation value in primary teeth regardless of the degree of root resorption, which has not affected this value or the accepted difference; however, DD and acceptable difference values are somehow affected by the degree of root curvature and canal size. We recommend adding acceptable difference as a criterion when considering pulpectomy treatment in primary teeth.

## 1 INTRODUCTION

It is very important to understand the details of the anatomy of the apex area to get a successful endodontic treatment. The importance of precising the position of the apical foramen (AF) increases in primary teeth, where overestimation during the endodontic procedure may hurt the permanent bud (Alafandy, 2018; Anandakrishna et al., 2017). The apex in primary teeth does not have the same constriction area as the apex in permanent teeth; therefore, the possibility of going beyond this wide AF is high in deciduous teeth, and this probability rises by the lifetime of the primary tooth due to the physiological resorption of the root, which results in continuous changes in the apex area. In the newly completed roots of the primary teeth, the AF is located near the anatomic apex (AA) of the roots (Waterhouse et al., 2011), or they may match each other, so in this case, AF locates in the same position of the AA. The physiologic resorption of the roots starts at the site that is closest to the permanent successor, so the resorption of primary incisors and canines starts at the lingual surfaces of the roots in the apical third (Harokopakis‐Hajishengallis, 2007). However, resorption in primary molars begins on the inner surfaces of the roots near the interradicular septum. As resorption progresses, the AF may not correspond to the AA of the root but positioned coronal to it. Subsequently, the radiographic establishment of the root canal length may be challenging (Waterhouse et al., 2011). The distance between the position of AA and AF is called the difference distance (DD), and it is changing continuously due to the root resorption in primary teeth. Thus, this difference varies widely and unpredictably from one root to another, and here is the dilemma that causes difficulties in evaluating working length in primary teeth.

In 2015, Manotas et al.'s study revealed that there was a relationship between the anatomical position of the apex and the AF, which must be taken into account by the dentist and the specialist during establishing the length of work in bicuspid teeth (Manotas et al., 2015).

The size of the root canals as well as the position of the AF is continually altered, making it difficult to determine the exact location of AF (Goerig & Camp, 1983; Kielbassa et al., 2003). So, this point needs to be studied more to see if there is a relation between the DD and the canal size in deciduous teeth.

Usually, to avoid going beyond AF in endodontic treatment in primary teeth, files are adjusted to stop 1 or 2 mm short of the radiographic apex of the canal (Barasuol et al., 2021; Bharuka & Mandroli, 2016), because with estimation errors within these values, the files would not exceed the root apex (Basso et al., 2015), or even harm the permanent bud. Therefore, in this research, we adopted this value (2 mm) to consider DD between the AF and the AA an acceptable difference (AD) if it is 2 mm or less, while it is unacceptable difference (UD) if it is more than 2 mm, and this may clarify the difference acceptance (DA) concept, which is the tolerance in the difference value between the actual and the estimated root canal length. The accepted value differs between permanent and primary teeth. Practitioners used to subtract 0.5 mm from radiographic root canal length in permanent teeth and 1–2 mm in primary teeth (Goerig & Camp, 1983) because it is taken for granted that the DD between AF and AA increases in deciduous teeth due to root resorption; but is this information accurate in all primary teeth? Does DD always increase when root resorption increases? Is the DD value affected by the size of the canal or even by the degree of root curvature?

*The aim* of this research was to study the position of the AF in relation to the AA by studying the DD and DA in root canals of primary teeth and to study the effect of canal sizes, root resorption degrees, and root curvature degrees on DD and DA.

## 2 MATERIALS AND METHODS

The manuscript of this laboratory study has been written according to the Preferred Reporting Items for Laboratory studies in Endodontology (PRILE) 2021 guidelines (Nagendrababu et al., 2021).

As the study used teeth that were previously extracted and were obtained in a manner that subjects could not be identified from the beginning, the need for informed consent was waived by the Research Ethics Committee, Scientific Research & Post Graduate Studies, Arab International University, Syria (Ref: Project No. 7‐1‐31‐10‐2021).

### 2.1 Sample

This study was conducted on 180 root canals of 60 extracted primary teeth, 12 first and five second upper molars, and 23 first and 20 second lower molars. The extracted teeth were collected from different dental clinics in Damascus city, and they were all extracted due to deep caries and pulpal involvement.

**Figure 1:**
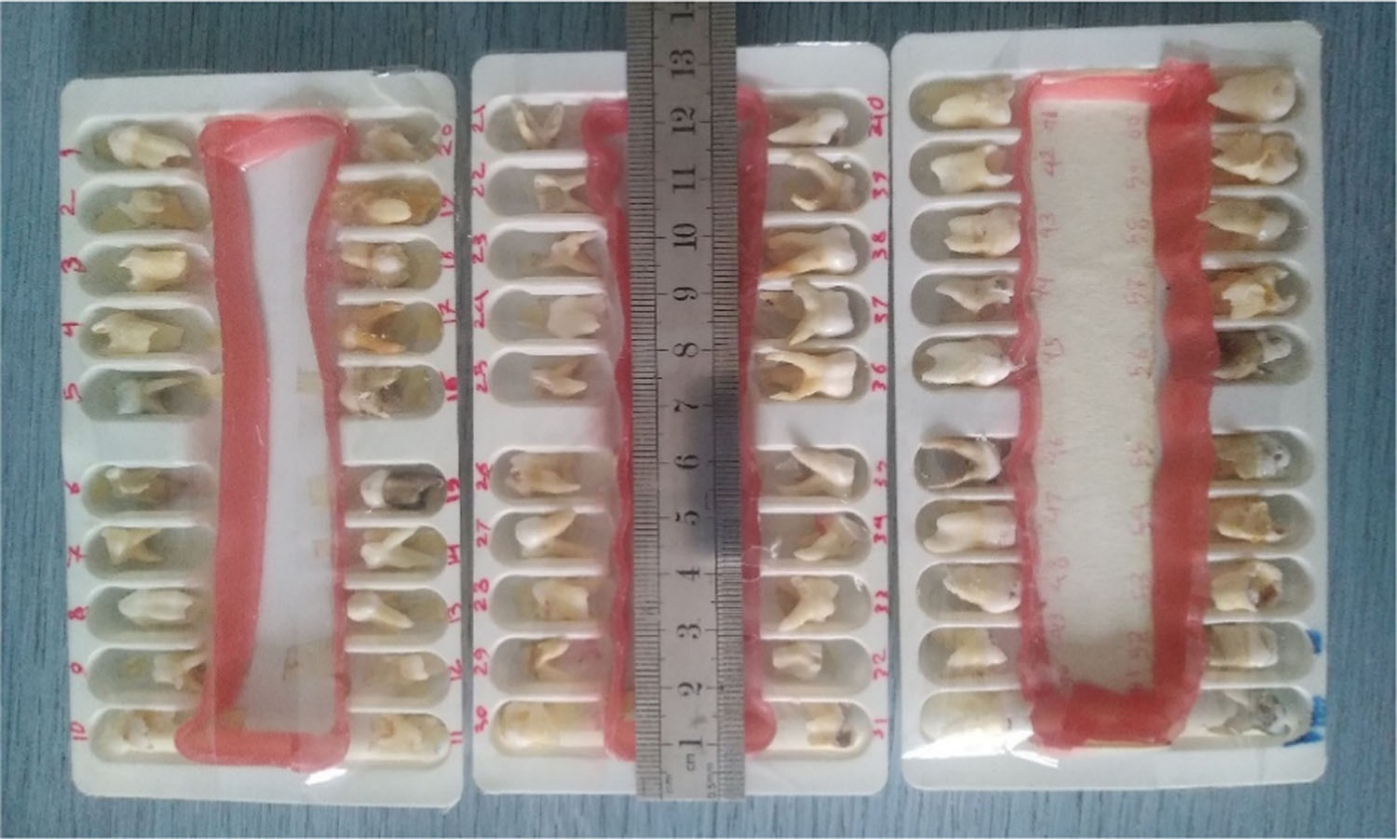
*The sample of research*. One hundred and eighty root canals from 60 extracted molars with root resorption half or less.

The upper canals were 43 (30 of first molars and 13 of second molars), while the lower canals were 137 (77 of first molars and 60 of second molars) (Figure 1).

The studied root canals were distributed as follows:

- *Seventy‐seven* canals of *first lower molars* (L1); 22 mesiobuccal (mb‐L1), 18 mesiolingual (ml‐L1), 14 distolingual (dl‐L1), 15 distobuccal (db‐L1), and 8 distal (d‐L1).
- *Thirty* canals of *first upper molars* (U1); 11 mesiobuccal (mb‐U1), 8 distobuccal (db‐U1), and 11 palatal (p‐U1).
- *Sixty* canals of *second lower molars* (L2); 15 mesiobuccal (mb‐L2), 16 mesiolingual (ml‐L2), 14 distolingual (dl‐L2), 11 distobuccal (db‐L2), and 4 distal (d‐L2).
- *Thirteen* canals of *second upper molars* (U2); 5 mesiobuccal (mb‐U2), 4 distobuccal (db‐U2), and 4 palatal (p‐U2).

### 2.2 Exclusion criteria

Canals with resorption of more than half of the root or calcified canals were excluded from this study.

### 2.3 Data measurements

**Figure 2:**
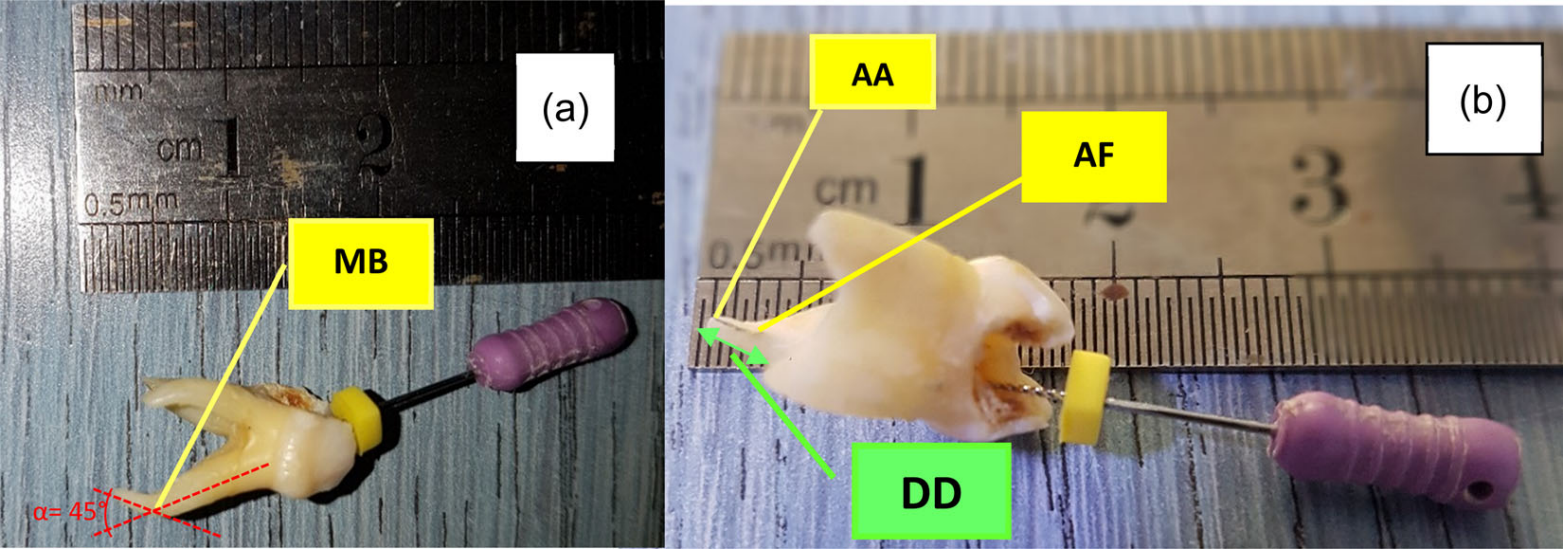
*Locating of apical foramen (AF) and anatomic apex (AA)*. The mesiobuccal root of the first upper primary molar with mild resorption, severe curvature α = 45° and fine canal size (a), difference distance between AF and AA in the same root (mesiobuccal canal) (b).

Using K‐files, each root canal was measured twice from a very specific point on the crown. The first measure was the full root length to locate the position of the AA by inserting a k‐file inside the canal to the tip of the root, while the second one was the working canal length to locate the AF position by introducing a k‐file into the canal until its tip emerged through the AF (Figure 2).

Different sizes of master K‐files, from 6 to 40, were used to adapt to different canals. Dental Loup Magnifier LED (×3.5 magnification, 420 mm marking distance, WALLY SKY) was used to visualize the tip of the file. Then the DD between the position of the AF and the AA was calculated. The measurement of each canal was repeated three times, then the mean measure was registered.

For measuring the root angle, the Schneider method was used. First, a line is drawn parallel to the long axis of the canal, in the coronal third; a second line is then drawn from the AF to intersect the point where the first line left the long axis of the canal. The Schneider angle is the intersection of these lines. If the angle is less than 5°, the canal is straight; if the angle is 5–20°, the canal is moderately curved; and if the angle is greater than 20°, the canal is classified as a severely curved canal (Balani et al., 2015; Patil et al., 2020), (Figure 2a).

To avoid bias, three doctors evaluated the classification of the canals. For each canal, the mean number of the three measurements was adopted.

### 2.4 Groups

The DD was classified according to the accepted difference in the endodontic treatment in primary teeth into two groups:

- Group 1; UD ( > 2 mm).
- Group 2; AD ( ≤ 2 mm).

The sample, which consists of 180 canals, was distributed according to:

I. *Resorption degree* into three groups as it appears in the radiograph (Alafandy, 2018):

- Group 1: 40 (22.2%) severe; resorption of half of the root.
- Group 2: 70 (38.9%) moderate; resorption of one‐third of the root.
- Group 3: 70 (38.9%) mild; resorption of one‐fourth of the root or less.

II. *Root curvature* into three groups:

- Group 1: 10 severe; the angle of root curvature >20°.
- Group 2: 46 moderate (25.6%); the angle of root curvature 5–20°.
- Group 3: 124 (68.9%) mild; the angle of root curvature <5°.

III. *Canal size* into three groups:

- Group 1: 99 (55%) fine canals (file size 6–10).
- Group 2: 64 (35.6%) medium canals (file size 15–25).
- Group 3: 17 (9.4%) wide canals (file size 30–40)

### 2.5 Statistical analysis

The raw data were processed using IBM SPSS Statistics 26. Descriptive statistics and nonparametric tests, the Kruskal–Wallis, the Mann–Whitney *U*, and the *χ* ^2^ tests as well as Spearman's correlation were implemented to achieve results. A *p* value of <.05 was considered significant at a 95% confidence level.

Illustrative images included in this manuscript were acquired using a Samsung Galaxy S7 edge, 12MP Camera (USA5.5 Display, 4 GB RAM, 32GB storage, microSD slot, 3600 mAh Battery). Images were put to explain the methods and materials followed in this research.

## 3 RESULTS

The DD varied between 0 and 6 mm. The count and percent of canals according to the DD value are presented in Table 1.

Statistics of the sample is shown below in Table 2.

**Table 1:** Distribution of canals according to DD.

Difference Distance (DD)	0.00	0.50	1.00	1.50	2.00	2.50	3.00	3.50	4.00	4.50	5.00	6.00	Total
Count of canals	62	35	28	15	12	8	5	3	5	2	3	2	180
Percent of canals	34.4%	19.4%	15.6%	8.3%	6.7%	4.4%	2.8%	1.7%	2.8%	1.1%	1.7%	1.1%	100.0%

### 3.1 Effect of variables on DD

**Table 2:** Descriptive statistics for difference distance (DD).

Statistics		
Difference distance		
*N*		
Valid		180
Missing		0
Mean		1.0750
Std. error of mean		0.09805
Median		0.5000
Mode		0.00
Std. deviation		1.31544
Variance		1.730
Range		6.00
Minimum		0.00
Maximum		6.00
Sum		193.50

**Figure 3:**
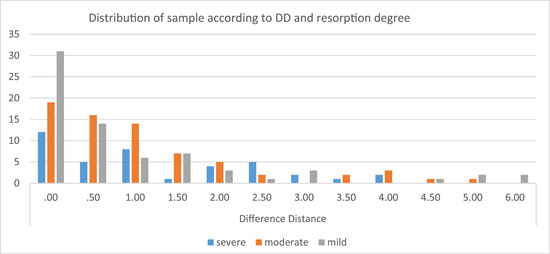
The distribution of the sample according to the difference distance (DD) value and resorption degree.

*1. Effect of root resorption degree*: The distribution of the sample according to the DD value and resorption degree is shown below (Figure 3). Using the Kruskal–Wallis test, a *p* value of .114 was obtained, which means that there was no significant difference between the DD value in the three groups of resorption degree.

**Figure 4:**
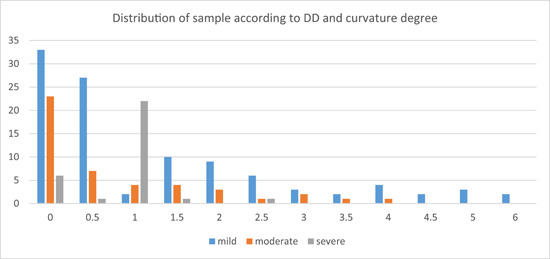
The distribution of the sample according to the difference distance (DD) value and curvature degree.

*2. Effect of root curvature degree*: The distribution of the sample according to the DD value and curvature degree is shown below (Figure 4). Using the Kruskal–Wallis test, a *p* value of .014 (*p* < .05) was obtained, which means that there was a significant difference between the three groups of curvature degree.

Therefore, the Mann–Whitney *U* test was done, and the *p* value was .503 when studying DD in severe and moderate groups, so there was no significant difference. On the other hand, *p* was less than .05 in the analysis of severe and mild groups (*p* = .049 < .05) and moderate and mild groups (*p* = .016 < .05), which means that there was a significant difference. It was observed that the mean rank of the mild group (97.73) was higher than that of the severe group (64.85) and the moderate group (76.58), which means that the mean of DD was higher in the mild curvature degree group than other groups.

**Figure 5:**
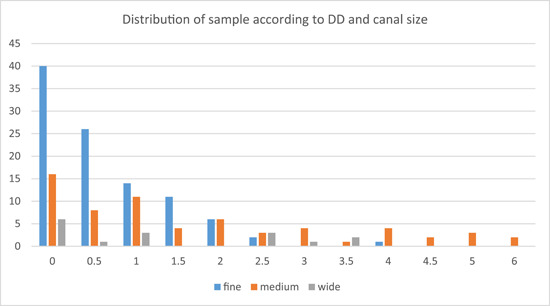
The distribution of the sample according to the difference distance (DD) value and canal size.

*3. Effect of canal size*: The distribution of the sample according to the DD value and canal size is shown below (Figure 5). The Kruskal–Wallis test was conducted and resulted in a significant *p* value of .000 < .05, which means that there was a significant difference between the three groups of canal size.

Therefore, the Mann–Whitney *U* test was done. The *p* value was .073 when studying DD in the fine and wide groups, and the *p* value was .510 when studying DD in the medium and wide groups, which means there was no significant difference in previous groups.

On the other hand, the *p* value was .000 when studying DD in the fine and medium groups, so there was a significant difference in DD value between the fine and medium canal size groups. The mean rank of the medium group (109.27) was higher than that of the fine group (76.71), which means that the DD was bigger in the medium canal size group.

### 3.2 Effect of variables on DA

**Figure 6:**
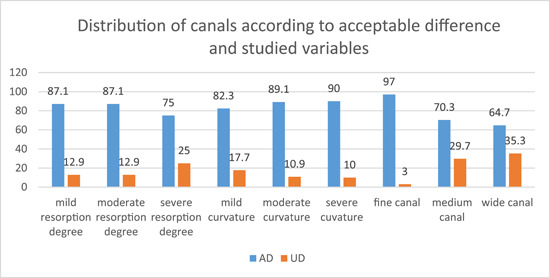
The distribution of canals according to acceptance difference in groups of each variable.

The percentages of *AD* and *UD* were 84.4% and 15.6%, respectively. The distribution of canals according to DA in groups of each variable is illustrated in Figure 6.

1. *Effect of root resorption degree*: The *χ* ^2^ test resulted in *p* = .174 > .05 with no significant difference between the DA in the groups.
2. *Effect of root curvature degree*: The *χ* ^2^ test resulted in *p* = .483 > .05 with no significant difference between the DA in the groups.
3. *Effect of root canal size*: The *χ* ^2^ test resulted in *p* = .000 < .05 with significant difference between the DA in the groups. It was clear that the fine canal group has the highest percentage of AD compared with the medium and wide canal groups.

The Spearman test revealed that the *p* value was .000, and the correlation was significant at the 0.01 level. The correlation coefficient was −0.381, which means that there was a negative weak correlation between canal size and DA. When canal size is wider, the difference will tend to be unacceptable.

## 4 DISCUSSION

To the best of our knowledge, there was no published research that studied the DD *value* in *primary* molars until the date of doing this research. Thus, the results of the current study were compared with similar studies performed on permanent teeth.

In this study, DD varied from 0 to 6 mm, and the percentage of AD was 84.4%. This *high* percentage *supported* the standard expected value of DD (2 mm), which used to be adopted by practitioners in endodontic treatment in primary teeth. Yet 15.6%, which was the percentage of the canals with *DD > 2 mm*, was a considerable percentage and should be taken in mind because overestimation may cause severe pain to the child, so the dentist may lose his cooperation; moreover, files could harm the permanent bud. So dentists should *not* take for granted 2 mm as a stable DD value and should use more accurate methods to determine the working length, such as apex locator (Alafandy, 2018; Kayabasi & Oznurhan, 2020).

The percentage of root canals with DD of 0 mm was 34.4%, and this is close to the result found by Martos et al., which was 40% in maxillary premolars (Martos et al., 2009). However, it is less than the percentage found by Manotas et al. in maxillary premolars with mature apices, which was 51% (Manotas et al., 2015).

The *mean* distance between the AF and the AA was *1 mm*, in spite of that, the maximum DD was 6 mm. This result may be explained by the high percentage of canals with DD ≤ 1 mm, which was 69.4% in this research.

The range of DD was between 0 and 6 mm. This *range* was so wide compared with that found in other studies in permanent teeth. It was between 0.43 and 1.05 mm in Wolf et al.'s research in the permanent maxillary and mandibular first and second molars (Wolf et al., 2017). Similarly, in Ayranci et al.'s research, DD was between 0.271 and 0.519 mm (Ayranci et al., 2013). So, it was noticeable that the variation of the DD value was less in the permanent teeth compared with the primary teeth. This result does make sense because continuous and unequal resorption in the two sides of the primary root may result in this great variation (0−6 mm) in the current study.

Although statistical analysis revealed no effect for the *degree of resorption* on DD and DA, the percentage of AD in the *mild and moderate* groups was prominently high (87.1%), while it was less in the severe resorption group (75%). Unpredictably, the highest values of DD (4.5–6 mm) were not registered in a canal with severe root resorption. This result may *change* the traditional concept, which assumes a positive correlation between the degree of resorption and the DD value.

**Figure 7:**
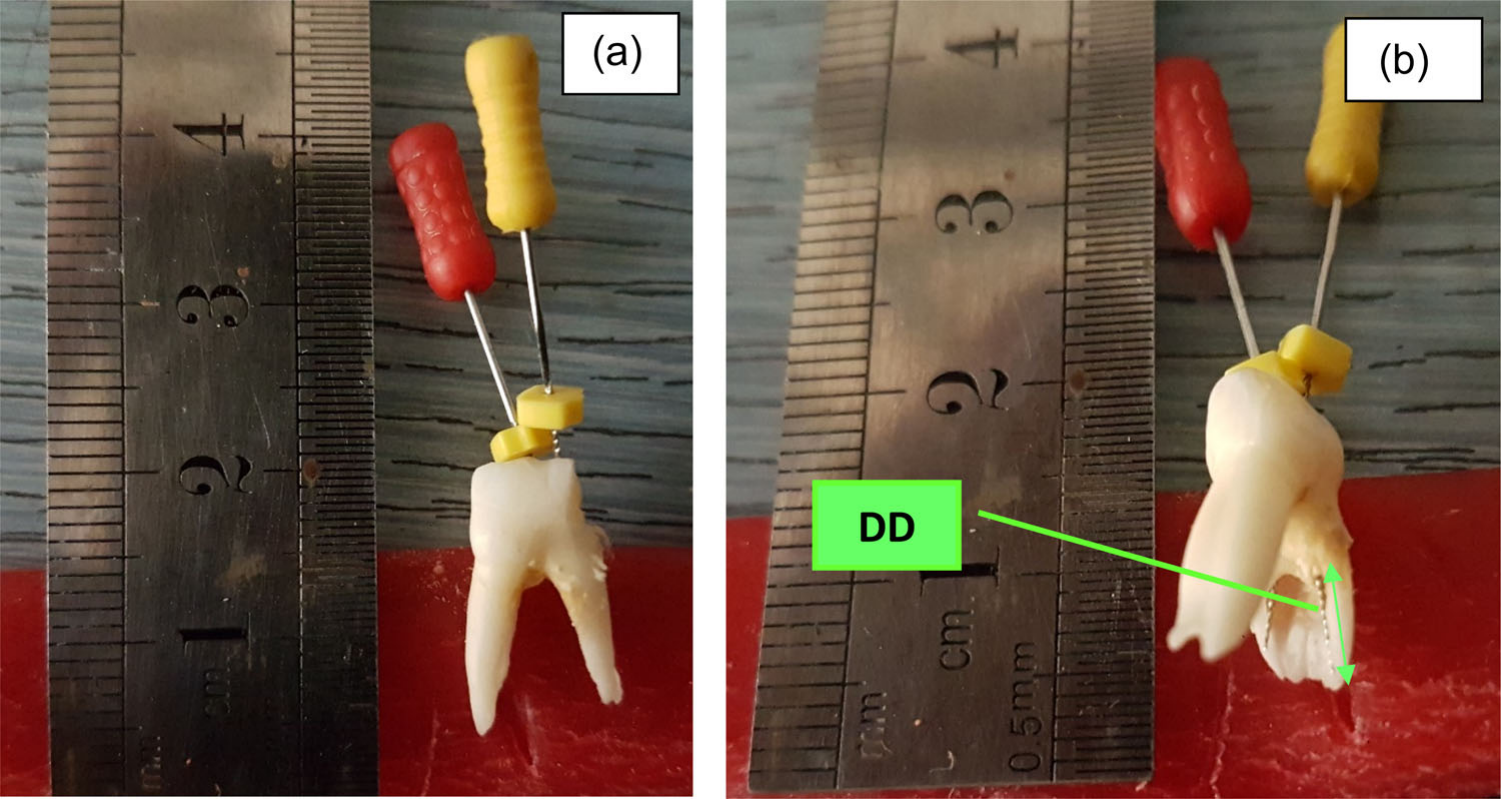
*Difference of root resorption degree between the out and inside aspects of the primary root*. Inserting K‐files in the distobuccal and distolingual canal of the first lower primary molar with moderate resorption (as it appears in the radiograph) (a), the difference distance (DD) is more than 4 mm in the same root because the resorption degree is more than half of the root in the inside aspect (b).

The primary teeth undergoing resorption showed smooth extensive and predominantly regular areas reflecting the slow ongoing physiologic process (Sreeja et al., 2009). Physiological resorption (PhR) occurs only at the apex of the primary teeth having a horizontal or *slightly* oblique pattern, while pathological root resorption (PaR) could be internal or external. The external resorption was classified into three categories: lateral, apical, or cervical (Gherghe et al., 2020). In this research, we could not classify root resorption into PhR or PaR according to the clinical and radiographic diagnosis of each case because there were no available data for each extracted tooth. However, according to the previous information, the type of lateral or severe oblique resorption may be PaR and could explain the big DD values (Figure 7) in roots with mild or moderate resorption degrees.

Resorption degree is one of the most important criteria for making the right decision in many pedodontic treatments, especially in endodontic treatment (Neuhaus & Kühnisch, 2019). However, root resorption is usually evaluated by radiographic image and does not consider the differences of resorption degree of each side of the root. It is almost impossible to define an accurate AF position in primary teeth depending on a radiographic image only due to asymmetric resorption patterns of the primary roots (Mokhtari et al., 2017), which may be invisible on two‐dimensional X‐ray (Figure 7).

Radiographic assessment of small areas of resorption is difficult particularly in cases where resorption occurs on the buccal or lingual aspects of the root (Anandakrishna et al., 2017). The permanent bud is located closer to the inner or lingual side of the primary molars or anterior teeth. This leads to a difference in resorption levels between the outer and the inner side of the root. Pathological resorption may increase this difference, and thus a considerable DD could be seen between the AA and the AF.

Studying the effect of the *root curvature degree* on DA revealed no significant difference between the three groups of curvature degree. However, there was a significant difference in DD between those groups. It was noticeable that the *highest* values of DD, which were between 4.5 and 6 mm, were all recorded in the *mild* curvature group. Analysis revealed that *the mean of DD is bigger in the mild curvature degree group* than in other groups. So DD seemed to be affected by root curvature. *The smaller the curvature is, the greater the DD is*.

This result may be explained by the oblique type of resorption, which leads to a bigger difference between the AA and the AF when the curvature of the root is mild, but when it is moderate or severe, the DD may become lower (Figure 5).

When studying the *effect of canal size*, it was noticeable that the highest values of DD (between 4.5 and 6 mm) were all recorded in the medium file size group. The significant difference in DD value was between the fine and medium canal size groups. DD was smaller in the *fine* canal size group, and this was confined to the percentage of AD in this group, where it was the highest (Figure 6).

The correlation coefficient was −0.381, which means that there was a weak negative correlation between canal size and DA. *When canal size was wider, the difference tended to be unacceptable*. Usually, canal size is wider in the young primary tooth. Internal resorption also causes canal widening, which is often a pathological resorption. Sometimes a physiological internal resorption in the late life of the primary tooth may cause canal widening (Waterhouse et al., 2011).

Thus, it is useful to take in mind the size of the canal when determining the working length in primary teeth; if it is 15 or more, then DD would be unacceptable.

Unfortunately, we could not compare our variables' results with other studies because there was no available research that studied the same variables.

### 4.1 The clinical importance of this research

In a nutshell, DD may not be discoverable by radiograph (Ricucci & Langeland, 1998). Many studies recommended the use of an electronic apex locator in primary teeth regardless of the stage of root resorption (Adriano et al., 2019; Alafandy, 2018; Angwaravong & Panitvisai, 2009; Kayabasi & Oznurhan, 2020; Nellamakkada et al., 2020; Oznurhan et al., 2015; Ricucci & Langeland, 1998). So if we predict *DD* by using a reliable apex locator and good radiograph, we will save a lot of effort in doing unsuccessful endodontic in primary teeth, especially if we take in mind the studied variables in the current study.

Finally, we *suggest* modifying the traditional radiographic root resorption classification to a new one that considers the unequal sides of root resorption.

Besides that, we suggest doing future research to study the relation between the DD value and root length and the success of endodontic treatment in primary teeth.

## 5 CONCLUSION

DD has a wide variation value in primary teeth regardless of the degree of root resorption, which has not affected this value or the accepted difference. However, DD and AD values are somehow affected by the degree of root curvature and canal size. We recommend adding AD as a criterion when considering pulpectomy treatment in primary teeth.

## AUTHOR CONTRIBUTIONS

1

Ansam Shafik Alafandy designed the study protocol, performed the in vitro experiments, and wrote the manuscript text. Ansam Shafik Alafandy and Ramah Eimad Makieh analyzed the data.

## CONFLICT OF INTEREST STATEMENT

The authors declare no conflict of interest.

## Data Availability

The data that support the findings of this study are available on request from the corresponding author. The data are not publicly available due to privacy or ethical restrictions.
